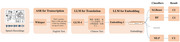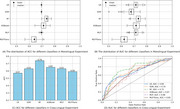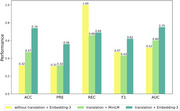# An Automatic and Speech‐based Cross‐Lingual Classification Framework for Early Screening of Cognitive Impairment

**DOI:** 10.1002/alz70856_099314

**Published:** 2025-12-24

**Authors:** Yue Wu, Yining Liao, Keyan Yu, Lele Chen, Zhuonan Wei, Lin Hu, Gaigai Lu, Hui Chen, Guanxun Cheng, Kai Wang, Xiang Fan

**Affiliations:** ^1^ Peking University Shenzhen Hospital, Shenzhen, Guangdong, China; ^2^ The Chinese University of Hong Kong – Shenzhen, Shenzhen, Guangdong, China; ^3^ Dongguan University of Technology, Dongguan, Guangdong, China

## Abstract

**Background:**

The use of speech data for distinguishing cognitive impairment (CI) is efficient and convenient for early screening of potential AD. However, few studies have developed available automated frameworks with the external cross‐lingual Chinese validation.

**Method:**

This study utilized speech data from the Cookie Theft description task, employing the ADReSSo dataset and the local Chinese dataset of the STAR cohort. We constructed an automated framework for CI screening, leveraging AI methods, including ASR, LLMs, and multiple types of machine learning classifiers. We used datasets in multiple languages and addressed the issue of language inconsistency.

**Result:**

Our framework achieved 74% in accuracy and 75% in AUC in the external cross‐lingual Chinese validation experiment. We conducted an ablation study to demonstrate the necessity of each module within the framework.

**Conclusion:**

The proposed framework provides fully automated assessments in distinguishing CI, making it highly beneficial for large‐scale early screening and self‐testing.